# Phasmid species that inhabit colder environments are less likely to have the ability to fly

**DOI:** 10.1002/ece3.10290

**Published:** 2023-07-20

**Authors:** Zachary Emberts

**Affiliations:** ^1^ Department of Integrative Biology Oklahoma State University Stillwater Oklahoma USA

**Keywords:** flight, insects, macroevolution, Phasmatodea, temperature, wind speed

## Abstract

A vast majority of insects can fly, but some cannot. Flight generally increases how far an individual can travel to access mates, enables the exploitation of additional food resources, and aids in predator avoidance. Despite its functional significance, much remains unknown about the factors that influence the evolution of flight. Here, I use phylogenetic comparative methods to investigate whether average annual temperature or wind speed, two components of the flying environment, is correlated with the evolution of flight using data from 107 species of stick and leaf insects (Insecta: Phasmatodea). I find no association between wind speed and flying ability in this clade. However, I find that colder temperatures are associated with the lack of flying ability. This pattern may be explained by the additional metabolic costs required for insects to fly when it is cold. This finding contradicts previous patterns observed in other insect groups and supports the hypothesis that cold temperatures can influence the evolution of flight.

## Graphical abstract

Here, I find that phasmid species that inhabit colder environments are less likely to have the ability to fly. This finding contradicts previous patterns observed in other insect groups and supports the hypothesis that cold temperatures can influence the evolution of flight.

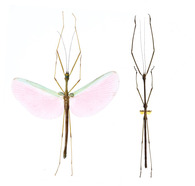

## 1 INTRODUCTION

The ability for insects to fly comes with many benefits (Zera & Denno, 1997). Most notably, flight can help individuals travel further, which enables access to additional resources (Zera & Denno, 1997). Moreover, the innovation of flight in insects was followed by a major radiation in the late Paleozoic Era, leading many to describe flight as having a key role in their evolutionary success (Kingsolver & Koehl, 1994; Wagner & Liebherr, 1992; Zera & Denno, 1997). Despite the ecological and evolutionary benefits of flight, some insects are unable to fly (Roff, 1994), and it is not completely clear why some (Pterygota insect) lineages are capable of flight while others are not.

Given that the ancestor of Pterygota insects was capable of flight, studies investigating variation in flying ability within (Pterygota) insects have largely focused on testing hypotheses that can explain how flying ability is secondarily lost (Leihy & Chown, 2020; Roff, 1990). One of the most discussed and supported hypotheses is the stable environments hypothesis (Roff, 1990; Wagner & Liebherr, 1992). This hypothesis proposes that when environments are persistent and stable, the evolution of flightlessness occurs because there is relaxed selection for rapid dispersal (i.e., a benefit of flight; Darlington, 1943; Roff, 1990). For example, orthopterans found in woodland habitats (where there should be relaxed selection for dispersal; Southwood, 1962) are more likely to be flightless (Roff, 1990). The stable environment hypothesis is clearly important (Roff, 1990), but other factors likely influence the evolution of flight as well (i.e., the flight evolution hypotheses are not mutually exclusive).

Another hypothesis, one that has not received as much attention, is that the flying environment has a role in shaping the evolution of flight. Both the ability to fly and the act of flying itself have energetic costs (Dudley, 2000). Moreover, some of these costs can be influenced by the environment in which flight occurs (e.g., Hennessy et al., 2021; Kenna et al., 2021; Niitepõld et al., 2009). Thus, if species persist in environments where flight is inhibited (because of the energetic costs required to fly), then there should be selection against investing resources into the morphological components required for flight (e.g., wings and flight muscles). Instead, individuals would likely gain a fitness advantage by allocating resources to other traits, such as those related to reproduction (Roff & Fairbairn, 1991). In such a scenario, selection should favor the evolution of flightlessness.

At least two factors related to the flying environment are hypothesized to influence the evolution of flight in insects, temperature and wind speed (Darlington, 1943; Downes, 1965; Leihy & Chown, 2020; McCulloch et al., 2019). Insects are generally considered to be ectotherms and therefore the temperature of their body and muscles is dependent on the temperature of the environment. Muscle temperature directly influences its activity (Coelho, 1991; Heinrich, 1975). As a result, flight in colder temperatures requires more energetic expenditure per wingbeat, increasing its energetic cost (Woods Jr et al., 2005). Even for species that are able to regulate the temperature of their flight muscles (i.e., heterothermic insects; Heinrich, 1970; Harrison et al., 1996), flight performance can be temperature dependent in some scenarios (Kenna et al., 2021).

In addition to cold temperatures, high wind speeds can also influence the cost of flight (Downes, 1965). Several field studies have shown that higher wind speeds decrease flying activity (Briers et al., 2003; Pinzauti, 1986; Vicens & Bosch, 2000). Moreover, flight performance can decrease as wind speed increases (Hennessy et al., 2021). Overall, these studies illustrate that both temperature and wind speed can influence the efficiency of flight and the frequency with which it is performed, which could ultimately influence its evolution.

**FIGURE 1:**
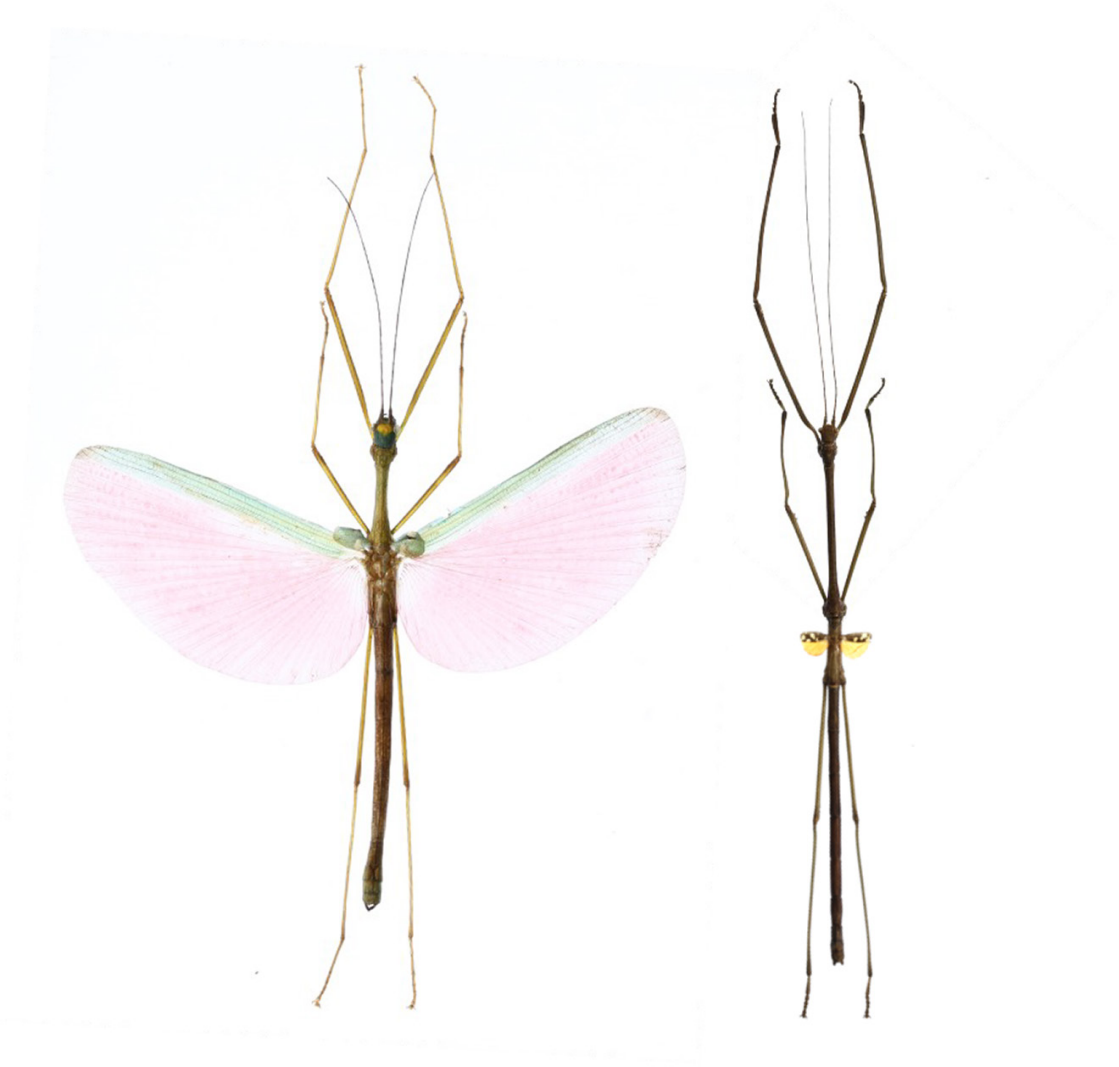
Many phasmids have long wings and are capable of flight, such as the *Marmessoidea* sp. pictured on the left. However, several other phasmids have reduced wings (such as the *Phaenopharos struthioneus* pictured on the right) or lack wings completely and are therefore unable to fly. Photographs of pinned specimens are not to scale.

Stick insects (Insecta: Phasmatodea) are a great clade to investigate hypotheses related to the evolution of flight (Figure 1). The ability to fly is incredibly variable in this group. Based on some estimates, approximately half of the species lack wings and are therefore incapable of flight (Zeng et al., 2020). Moreover, the evolution of flight in phasmids is thought to be fairly labile, with multiple evolutionary transitions between nonflying and flying states (Bank & Bradler, 2022; Forni et al., 2022; Whiting et al., 2003). The direction of these evolutionary transitions is debated. Some hypothesize that flight was lost multiple times (Stone & French, 2003; Trueman et al., 2004), while others hypothesize that flight was reacquired multiple times (Bank & Bradler, 2022; Forni et al., 2022; Whiting et al., 2003). Importantly, while the flight evolution hypotheses discussed above were originally proposed to explain the evolution of flight loss, they can also be expanded to include the re‐evolution of flight. For example, when environments are unstable, there should be increased selection for dispersal (Roff, 1990), and one mechanism by which dispersal can increase is through flight (Zera & Denno, 1997). Moreover, if there is selection for flight, a harsh flying environment may constrain its evolution. Finally, another great reason to use stick insects is that they have a largely cosmopolitan distribution (Simon et al., 2019) and can be found in a variety of habitats, spanning different temperatures and wind speeds. Combined, these factors enable me to rigorously test macroevolutionary predictions made by the high wind speed and low temperature hypotheses.

The low temperature hypothesis predicts that species that inhabit colder environments will be less likely to have the ability to fly. Therefore, as temperature decreases, so should flying ability (i.e., a positive correlation). The high wind speed hypothesis predicts that species that inhabit environments with higher wind speeds will be less likely to have the ability to fly. Thus, as wind speed increases flying ability should decrease (i.e., a negative correlation). To test these predictions, I integrate data on flying ability, the environment in which flight occurs (i.e., temperature and wind speed), and phylogeny for 107 species of stick insects. Then, I conduct phylogenetic logistic regressions to determine whether temperature and/or wind speed are associated with the evolution of flight.

## 2 METHODS

### 2.1 Tree and trait data

The main phylogeny (B2) reported in Bank and Bradler (2022) was used here. This tree included 515 taxa, two of which formed the out‐group. This study (Bank & Bradler, 2022) represented the most comprehensive taxon sampling of the group and captured approximately 47% of the generic diversity (243/517; Roskov et al., 2019). Their (Bank & Bradler, 2022) main time‐calibrated phylogeny is based on a constrained BEAST (Bouckaert et al., 2019; i.e., Bayesian inference) analysis using seven loci (three nuclear and four mitochondrial). The constraint was used to ensure that the relationships of major phasmid subclades were congruent with previous transcriptomic studies (Simon et al., 2019; Tihelka et al., 2020). In addition to their main tree, Bank and Bradler (2022) reconstructed two additional phylogenies using slightly different constraints (B1 and B3). Their main phylogeny (B2; Bank & Bradler, 2022) had the highest nodal support. However, all their phylogenies had relatively similar topologies and the relationships between the major lineages were strongly supported.

The Bank and Bradler (2022) phylogeny also had matching data on flying ability. Flying ability was determined by using wing size data collected from photographs and the literature. If the hind wings exceeded the fourth abdominal segment, then the species was considered fully winged (macropterous) and capable of flight (volant). Thus, this coding scheme denotes which phasmid species have long wings and which do not. Species without wings are certainly incapable of flight. Additionally, phasmids with small wings are unable to fly, but some can parachute or glide (Zeng et al., 2020). However, if a phasmid species has long wings, they are generally assumed to be capable of flight but this is not always the case (e.g. Bank & Bradler, 2022; Zeng et al., 2020, 2022). Here, I assume that all phasmids with long wings can fly. Then, I conduct simulations to explicitly test how sensitive the results are to this assumption (see Statistical analyses and results below). Bank and Bradler (2022) separately assigned flying ability for each sex. For this study, a species was considered capable of flight if males and/or females had long wings (Dataset S1).

rGBIF was then used to query the Global Biodiversity Information Facility (i.e., GBIF) for occurrence data for all 515 taxa on June 23, 2022 (GBIF Occurrence Download, 2022). This resulted in 13,821 raw occurrences (GBIF Occurrence Download, 2022). Raw occurrences were then filtered for unique observations. This step removed duplicated data points and reduced the number of occurrences to 11,860 (Dataset S2). Duplicate data points can occur when two individuals of one species are found in the exact same location and each individual gets recorded separately. Removing these duplicates should help prevent location biases from influencing downstream calculations.

Monthly temperature (°C) and wind speed (m/s) data at a resolution of ~20 km^2^ (i.e., 2.5 min) was then downloaded from WorldClim (Fick & Hijmans, 2017). WorldClim uses mean temperature data from 20,268 locations and average wind speed data from 10,149 locations (collected between 1970 and 2000) and models these variables across the globe (Fick & Hijmans, 2017). Estimates between observed and expected values are highly correlated for mean temperature (ρ = .996), but the model accuracy is lower for wind speed (ρ = .759; Fick & Hijmans, 2017). Mean temperature and wind speed data were then extracted for each phasmid occurrence point using the r package raster (version 3.5; Hijmans, 2022). To get yearly average temperature and wind speed data, the monthly averages were summed and divided by 12. If temperature and wind speed could not be acquired for an occurrence point, then the occurrence point was removed. This resulted in a dataset with 11,748 occurrence points (Dataset S3).

Following other studies (e.g., Howard et al., 2019), species with less than three unique occurrence points were also removed. Increasing the number of occurrences per species should help increase the chance that the average environment in which a species inhabits is accurately captured. Moreover, having more occurrence points per species should help reduce the impact that misidentified occurrence points potentially have on the averaged climate data. However, increasing the minimum number of occurrences per species will also reduce the number of species that will ultimately be included in the dataset. Using a cutoff of three unique occurrence points per species attempts to maximize the number of species included in the dataset, while also trying to accurately capture the average temperature and wind speed that each species inhabits. This step resulted in a dataset of 11,644 unique observations from around the world (Figure S1). The average annual temperature and wind speed data for each species was then obtained by taking the mean of all specimens per species. Out‐group taxa and duplicated species were not included in any of the final datasets.

Another publicly available dataset was also considered. This dataset included information on flying ability and body size for 129 phasmid taxa and was coupled with a phylogenetic hypothesis (Zeng et al., 2020). However, after following the methodology outlined above, only 40 species had at least one occurrence point and only 29 species had at least three. Despite the small sample size, the inclusion of different taxa, and a different phylogenetic hypothesis, the results are congruent with those present in the main text (Appendix S1).

### 2.2 Statistical analyses

To test the hypotheses that wind speed and temperature influence the evolution of flight in stick insects, I conducted multiple phylogenetic logistic regressions (Ives & Garland, 2010) using phylolm (version 2.6.2; Ho & Ané, 2014) in R (version 4.2.1; R Core Team, 2022). Phylogenetic logistic regressions test whether the transitions to a given state (in this case, flying ability) is dependent upon another factor (in this case, temperature or wind speed). This method incorporates phylogeny and allows for the dependent variable to be binary and the independent variable to be continuous. Long wings and the presumed ability to fly were coded as “1”, while short and/or absent wings were coded as “0”. Thus, a positive correlation between flying ability and temperature would support the cold temperature hypothesis. Moreover, a negative correlation between flying ability and wind speed would support the high wind speed hypothesis.

I also tested the robustness of the results to (1) topological uncertainty, (2) the minimum number of occurrence points required per species, (3) incomplete sampling of the phasmid clade, (4) the assumption that all long‐winged phasmid species can fly, and (5) the possibility that the observed patterns could potentially be explained by elevation (as opposed to the ecological variables of interest, wind speed and temperature). To determine if the results were sensitive to topological uncertainty, I reanalyzed the main results using the other two publicly available phylogenies reported in Bank and Bradler (2022) (B1 and B3). To determine if the minimum occurrence point threshold (i.e., 3) influenced the results, I also reconducted the analyses on datasets in which (1) each species had at least 10 unique occurrence points and (2) each species had at least 20 unique occurrence points.

I then tested whether the main conclusions were driven by incomplete taxon sampling. I did this by subsampling ~50% of the species in the main phylogeny 100 times, resulting in 100 new trees and corresponding datasets (similar to Moreira et al., 2021). This subsampling was conducted by randomly dropping 53 tips from the tree with 107 species (107–53 = 54) using the drop.tip function in the R package ape (version 5.4; Paradis & Schliep, 2019). I then conducted two phylogenetic logistic regressions on each replicate. For the logistic regressions, flying ability was the dependent variable and average temperature or wind speed was the independent variable. I then summarized the direction of the coefficients from these phylogenetic logistic regressions (i.e., was it a positive or a negative relationship) and noted whether the relationship was statistically significant (*p* < .05). Simulations have shown that incomplete taxon sampling can reduce statistical power but rarely results in false positives (Ackerly, 2000). Thus, these subsampling analyses may have reduced statistical power, but they should not generate statistically significant results that contradict those based on more complete taxon sampling.

I also tested the robustness of the main results to ~10% error in the assumption that all phasmid species with long wings can fly. I did this by conducting another 100 replicates of the dataset. For each replicate, four random phasmid species with long wings (~10% of the long wing species) were assumed to be incapable of flight. I then conducted two phylogenetic logistic regressions on each replicate and summarized the results.

Finally, I investigated the degree to which incorporating elevation data influenced the main results. Specifically, I extracted elevation data for each occurrence point using the R package elevatr (version 0.4.4; Hollister, 2022). I then obtained the average elevation for each species by taking the mean of all specimens per species. Next, I conducted two additional phylogenetic logistic regressions. For the first logistic regression, flying ability was the dependent variable and both elevation and temperature were the independent variables. For the second logistic regression, flying ability was the dependent variable and both elevation and wind speed were the independent variables.

## 3 RESULTS

**FIGURE 2:**
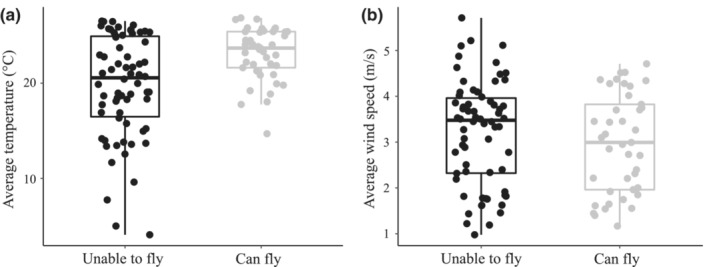
Box and whisker plot showing the (a) average yearly temperature and (b) average yearly wind speed at which 41 flying and 66 nonflying phasmid species are found. This figure does not correct for phylogeny. Boxes indicate the interquartile range for flying versus nonflying insects, whereas the whiskers indicate the range when excluding outliers.

**FIGURE 3:**
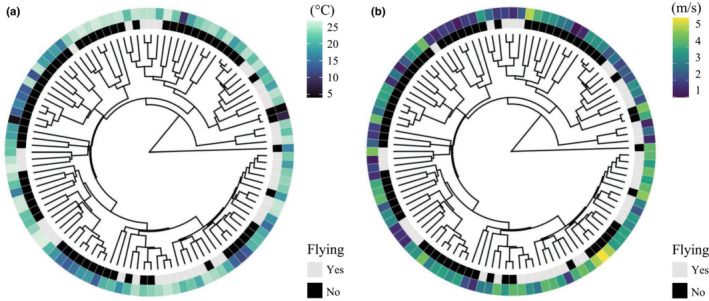
Environmental features and flying ability across phasmid species. In subfigure (a), the dark blue color on the outer ring (i.e., colder temperatures) is generally associated with the black color on the inner ring (i.e., inability to fly). However, some stick insects that are unable to fly can be found at warmer temperatures (i.e., sea foam green). This suggests that other factors, in addition to temperature, likely influence the evolution of flight in this clade. Given that the flight evolution hypotheses are not mutually exclusive, future studies should continue investigating additional factors that influence the evolution of flight in phasmids (e.g., stable environment hypothesis). In subfigure (b), wind speed (i.e., the outer ring) and flight (i.e., inner ring) appear uncorrelated. These phylogenies also visually reveal that there were likely several transitions between flight states across phasmids.

Of the 513 phasmid taxa investigated, 107 (~20%) had at least three unique occurrence points from which average annual temperature and wind speed could be calculated. Sixty‐six of those species were incapable of flight (62%), and 41 had long wings and were assumed to be able to fly (38%). Flight capable species were found in environments that had an average annual temperature of 23.1°C, whereas species incapable of flight were found in environments that had an average annual temperature of 19.7°C (Figure 2). Thus, colder temperatures were associated with the inability to fly (B2: estimated coefficient = 0.153, *z* = 2.242, *p* = .025; Figure 3). This pattern was consistent regardless of which phylogeny was used (B1: estimated coefficient = 0.163, *z* = 2.538, *p* = .011; B3: estimated coefficient = 0.164, *z* = 2.546, *p* = .011). Flight capable species were also found in environments that had an average wind speed of 3.0 m/s, while species incapable of flight were found in environments that had an average wind speed of 3.2 m/s (Figure 2). However, wind speed was not significantly associated with flying ability in phasmids (B2: estimated coefficient = −0.035, *z* = −0.189, *p* = .850; Figure 3). This result was consistent for all investigated phylogenies (Table S1).

The main dataset (results above) required there to be at least three unique occurrence points per species, a relatively small number of geographic occurrence points. Thus, I also tested whether increasing the minimum number of occurrence points required impacted the results. When I required a minimum of 10 occurrence points per species, the number of species included in the dataset was reduced to 59. Despite the reduced sample size, there was still a significant association between temperature and flying ability in phasmids (B2: estimated coefficient = 0.243, *z* = 3.156, *p* = .002). This pattern was consistent regardless of which phylogeny was used (B1: estimated coefficient = 0.239, *z* = 3.126, *p* = .002; B3: estimated coefficient = 0.241, *z* = 3.138, *p* = .002). Next, I required a minimum of 20 occurrence points per species. This reduced the sample size to 39 species. Again, despite a further reduction in sample size, there was still a significant association between temperature and flying ability in phasmids (B2: estimated coefficient = 0.384, *z* = 3.071, *p* = .002). This pattern was consistent regardless of which phylogeny was used (B1: estimated coefficient = 0.381, *z* = 3.044, *p* = .002; B3: estimated coefficient = 0.382, *z* = 3.046, *p* = .002). Wind speed, on the other hand, was never associated with flying ability in phasmids, regardless of the minimum number of occurrence points set per species (Table S1).

Even the largest dataset (*n* = 107) included only a fraction of the known phasmid species. Thus, I also tested the robustness of the main results to limited taxon sampling. I did this by randomly sampling ~50% (*n* = 54) of the species in the main tree 100 times and reanalyzing each replicate. When investigating the relationship between flight and temperature, all 100 of the replicates had positive coefficients, indicating that cold temperature is associated with the inability to fly. Seventy‐three of these relationships were statistically significant, while 27 were not. Most importantly, there were no instances in which the results from the subsampling analyses conflicted with those from full sampling. This suggests that the main result (i.e., a significant association between temperature and flying ability) is not driven by limited taxon sampling.

I also tested the robustness of the main results to potential error in the assumption that all phasmid species with long wings can fly. I did this by conducting 100 replicates of the main dataset and randomly assuming that four phasmid species with long wings were incapable of flight (i.e., ~10% of the flight capable species). Then, I reanalyzed the data. When investigating the relationship between flight and temperature, all 100 of the replicates had positive coefficients, indicating that cold temperature is associated with the inability to fly. Moreover, 90 of these relationships were statistically significant. When investigating the relationship between flight and wind speed, 99 of the coefficients were negative and one was positive. However, none of these relationships were statistically significant, concordant with the main results. Overall, these findings indicate that the main conclusions are robust, even if ~10% of long‐winged phasmid species are incapable of flight.

Finally, I tested whether the observed patterns were driven by elevation as opposed to the ecological variables of interest. Specifically, I reran the two main phylogenetic logistic regressions, but this time I also included elevation as a covariate. Even after taking elevation into consideration, the same patterns emerge. Phasmid species that inhabit colder environments are less likely to have the ability to fly (B2: estimated coefficient = 0.202, *z* = 3.379, *p* = .001; Table S2). However, wind speed is not associated with flying ability in phasmids (B2: estimated coefficient = −0.208, *z* = −1.210, *p* = .226; Table S3).

## 4 DISCUSSION

Whether or not an insect species can fly can have major ecological and evolutionary implications (Kingsolver & Koehl, 1994; Wagner & Liebherr, 1992; Zera & Denno, 1997). However, much remains unknown about the factors that influence the evolution of flight. Here, I investigated whether wind speed and temperature, two components of the flying environment, could explain variation in flying ability among stick insects (Insecta: Phasmatodea).

I found no evidence to suggest that wind speed was associated with the evolution of flight in this clade (Table S1, Appendix S1). There are several scenarios that could potentially explain why I did not find the predicted pattern—that higher wind speeds decrease flying ability (Downes, 1965). First, it is possible that wind speed only has a small influence on the evolution of flight in stick insects (as suggested by the estimated coefficients; Table S1). In which case, my sample size (*n* = 107 species) may not be large enough to detect such a small effect. Another possibility is that body size, shape, and/or weight may interact with wind speed to explain the evolution of flight. Previous studies have shown that body size and shape can influence flying ability (e.g. Boisseau et al., 2022). Thus, high wind speeds may only constrain the evolution of flight if species are large. However, the supplemental analyses (Appendix S1, Figure S2), which incorporate body size, do not support this conclusion. It is also possible that the high wind speed hypothesis is location dependent. For example, on islands, high wind speeds could increase the energetic cost of flight and increase displacement risk (e.g., by increasing the chance of dispersing into an inhospitable area, such as the open ocean; Darwin, 1859; Leihy & Chown, 2020). In such a scenario, both costs may work together to promote and/or maintain flightlessness. Another possibility is that the wind speed hypothesis is habitat dependent (Foster et al., 2021; McCulloch et al., 2019). For example, some phasmids may reside (and fly) in habitats that greatly reduce wind speed (e.g., the rainforest understory). In which case, the unobstructed wind speed 10 m above the ground likely has minimal impact on the energetic cost of their flight. Future work should continue to investigate these, and other, hypotheses.

The results also revealed that phasmid species that inhabit colder environments are less likely to have the ability to fly. This pattern held even when taking elevation into consideration (Table S2). Moreover, all the species in this study inhabiting environments that had average annual temperatures below 14°C were unable to fly (Figure 2), which further suggests that there is a temperature cutoff at which flight is not favorable. These findings support the hypothesis that cold temperatures can constrain the evolution of flight. Relatively few studies have explicitly tested predictions from this hypothesis. One of the most comprehensive studies investigating the evolution of flight loss (specifically on Antarctic, Arctic, and Southern Ocean islands) also found that temperature was significantly associated with flying ability (Leihy & Chown, 2020). But, Leihy and Chown (2020) found the opposite pattern predicted by the cold temperature hypothesis—species incapable of flight were more likely to be found at warmer temperatures. There are few potential reasons that my results contradict those in Leihy and Chown (2020). First, despite Leihy and Chown (2020) investigating the evolution of flying ability of island‐dwelling insect taxa, their dataset does not include any phasmid species. Thus, it is possible that the cold temperature hypothesis applies to phasmids but not to other insect groups. Another possibility is that warm temperatures only promote flightlessness on Antarctic, Arctic, and Southern Ocean islands (i.e., relatively cold places). However, at a broader geographic scale, cold temperatures may be influencing the evolution of flying ability. Nonetheless, the results presented here are the first to my knowledge to statistically support predictions made by the cold temperature hypothesis, highlighting that temperature (particularly cold temperature) may have an important role in explaining the evolution of flight in insects.

There are a few limitations associated with this study. First, this study assumes that all long‐winged phasmids can fly. However, species with long wings may be incapable of flight. For example, in stoneflies, the ability to fly appears to be lost before a reduction in wing size is observed (McCulloch et al., 2009). This should not be problematic, given that the main conclusions are robust to ~10% error in the assumption that all long‐winged phasmid species can fly. Another limitation of this study is that it only includes a fraction of the known phasmid diversity (~3%; 107/3411; Roskov et al., 2019). However, I used the most comprehensive phasmid phylogeny that was available. Moreover, the results were largely congruent across a range of sample sizes (*n* = 39, 59, and 107) and datasets (Appendix S1). Furthermore, when I randomly removed half of the species in the main dataset and reran the analyses (which I did 100 times), I found a statistically significant association between temperature and flying ability for a majority of the replicates (73%). Cold temperature was generally associated with the inability to fly in the remaining replicates as well, but these associations were non‐significant. These results are consistent with simulations which illustrate that incomplete taxon sampling can reduce statistical power but rarely results in false positives (Ackerly, 2000). Collectively, these findings indicate that the main result (i.e., a significant association between temperature and flying ability) is not driven by limited taxon sampling. Another potential issue is that I used average annual temperature and wind speed (as opposed to more fine scale data). For example, some phasmid species can live for multiple years, while others are thought to live for only a few months (e.g. Berger, 2004). In which case, datasets that include average temperature and wind speeds during the months that a phasmid species is in their adult form could potentially be more informative. However, despite using yearly averages, I was still able to detect a strong relationship between temperature and flying ability. Finally, it is important to note that I have identified a correlation between temperature and flying ability in phasmids. This finding supports the cold temperature hypothesis. However, other factors could hypothetically be contributing to this pattern. For example, it is possible that flying phasmids do not disperse to regions where it is cold. In which case, temperature may be restricting the ecological niche for flight‐capable species. Now that this pattern has been identified, future work should continue to investigate the degree to which both scenarios (ecology and evolution) are responsible for driving this pattern.

In summary, the ability to fly has many ecological benefits and is thought to have played a key role in the evolutionary success of insects (Kingsolver & Koehl, 1994; Wagner & Liebherr, 1992; Zera & Denno, 1997). However, not all insects can fly (Roff, 1994), and much remains unknown about the factors that influence the evolution of this trait (Leihy & Chown, 2020; Roff, 1990). Here, I found evidence to support the hypothesis that cold temperatures influence the evolution of flight in phasmids. Specifically, I found that species that inhabit colder environments are less likely to have the ability to fly. This may help explain why some insects are capable of flight while others are not.

## AUTHOR CONTRIBUTIONS

**Zachary Emberts:** Conceptualization (equal); formal analysis (equal); investigation (equal); methodology (equal); visualization (equal); writing – original draft (equal); writing – review and editing (equal).

## CONFLICT OF INTEREST STATEMENT

I have no conflicts of interest to declare.

## Supporting information


Dataset S1.

Dataset S2.

Dataset S3.

Dataset S4.

Dataset S5.

Dataset S6.

Dataset S7.

Dataset S8.

Dataset S9.

Dataset S10.

Data S1.

## Data Availability

All data, phylogenies, and R code needed to replicate my analyses is available in the supplementary material.
