# Supplementary material for: Phasmid species that inhabit colder environments are less likely to have the ability to fly
Source: Ecol Evol. 2023 Jul 20;13(7):e10290. doi: 10.1002/ece3.10290 (PMC10361346; doi:10.1002/ece3.10290)
Supplement: Supplementary file 11 — Data S1. [file ECE3-13-e10290-s007.pdf]

## Supplementary Information for

# Phasmid species that inhabit colder environments are less likely to have the ability to fly

**This PDF file includes**
Appendix S1.
Figure S1 and S2.
Table S1–S3.

**Other supplementary materials for this manuscript include the following (in separate files):**
Dataset S1. Phasmatodea assumed flying ability data from Bank and Bradler (2022).
Dataset S2. Occurrence and environmental data for species that match Datasets S6–8. This dataset includes duplicate occurrence points.
Dataset S3. Occurrence and environmental data for species that match Datasets S6–8, excluding duplicate occurrence points.
Dataset S4. Phasmatodea body size and assumed flying ability data from Zeng et al. (2020).
Dataset S5. Occurrence and environmental data for species that match Dataset S9.
Dataset S6. Main dated Phasmatodea phylogeny in nexus tree format, which is referred to as B2 in Bank and Bradler (2022).
Dataset S7. Dated Phasmatodea phylogeny in nexus tree format, which is referred to as B1 in Bank and Bradler (2022).
Dataset S8. Dated Phasmatodea phylogeny in nexus tree format, which is referred to as B3 in Bank and Bradler (2022).
Dataset S9. Dated Phasmatodea phylogeny in nexus tree format from Zeng et al. (2020).
Dataset S10. R code.

**Appendix S1. Cold temperature is associated with the lack of flying ability in stick insects, even when taking body size into consideration.**
I investigated the cold temperature and high windspeed hypotheses using another phasmid phylogeny that had corresponding data on flying ability (Zeng et al. 2020). This dataset also included information on body size, which allowed me to investigate whether the patterns observed and reported in the main text would be similar when including body size as a covariate.

## *Methods*

### DATA COLLECTION

The phylogeny used in Zeng et al. (2020) includes 129 phasmid taxa, with representatives from ~70% of the phasmid tribes. This time-calibrated Bayesian Inference phylogeny is based on a BEAST (Drummond et al. 2012) analysis using 4 loci (2 nuclear and 2 mitochondrial). Flying ability was determined by using wing and body size data collected from photographs and the literature (Zeng et al. 2020). Species without wings were considered incapable of flight. Winged taxa had a bimodal distribution for relative wing size, and species with wings that were 40% as long as their body size were considered to have long wings and assumed to be able to fly. Species with long wings were coded as “1”, while all others were coded as “0” (Dataset S4). Since male body size was going to be used in the analyses, only male wing size was considered when determining whether a species was considered capable of flight.

rGBIF was then used to query the global biodiversity information facility (i.e. GBIF) for occurrence data for all 129 taxa on July 14, 2022 (GBIF Dataset). This resulted in 11,449 raw occurrences (GBIF Dataset). Mean monthly temperature and windspeed data (at a resolution of ~20km$^2$) was then extracted for each phasmid occurrence point using the r package raster (version 3.5; Hijmans 2022). To get yearly average temperature and windspeed data, the monthly averages were summed and divided by 12. Duplicated data points and those for which temperature and windspeed could not be acquired were then removed from the dataset. This step reduced the number of occurrences to 9,732 (Dataset S5).

Previous studies (e.g. Howard et al. 2019), and our main analyses, remove species with less than 3 unique occurrence points. However, in this case, this decision would result in only 29 species being included in the final dataset (and only 12 of which had long wings). Thus, I reduced the number of occurrence points required to 1, which resulted in 40 species being included in the final dataset. Outgroup taxa were not included in this dataset.

### STATISTICAL ANALYSES

To test the hypotheses that windspeed and temperature influence the evolution of flight in stick insects I conducted multiple phylogenetic logistic regressions (Ives and Garland 2010) using phylolm (version 2.6.2; Ho and Ané 2014) in R (version 4.2.1; R Core Team 2022). First, I investigated whether an environmental variable (i.e. temperature or windspeed), body size, and their interaction influence the evolution of flight. For these analyses, I used $p > 0.15$ to identify whether the interactions were insignificant (to be more conservative, following Bursac et al. 2008), which they were. Thus, I conducted two additional phylogenetic logistic regressions in which the interaction term was not included (i.e. the interactions were dropped from the models).

## *Results*

Using the Zeng et al. (2020) dataset, I found that windspeed was not significantly associated with flying ability (estimated coefficient=-0.803, z=-1.693, p=0.090). However, I found that colder

temperatures were associated with the inability to fly (estimated coefficient=0.176, z=2.502, p=0.012; Figure S2). Thus, these results (which correct for body size) are concordant with the main results despite (1) the much smaller sample size, (2) the inclusion of different taxa, and (3) a different phylogenetic hypothesis. Additionally, in both models, I found that larger body size is associated with the evolution of flight in phasmids (as previously identified in Zeng et al. 2020; windspeed model: estimated body size coefficient=0.051, z=2.891, p=0.004; temperature model: estimated body size coefficient=0.074, z=3.308, p=0.001; Figure S2).

**LITERATURE CITED**


Bursac, Z., C.H. Gauss, D.K. Williams, D.W. Hosmer. 2008. Purposeful selection of variables in logistic regression. Source Code Biol. Med 3:1–8.

Drummond, A.J., M.A. Suchard, D. Xie, A. Rambaut. 2012. Bayesian phylogenetics with BEAUti and the BEAST 1.7. Mol. Biol. Evol 29:1969–1973.

GBIF.org. 14 July 2022. GBIF Occurrence Download (doi: https://doi.org/10.15468/dl.tvuhsv)

Hijmans, R. 2022. raster: Geographic Data Analysis and Modeling. R package version 3.5-29.

Ho, L.S.T., C. Ané. 2014. A linear-time algorithm for Gaussian and non-Gaussian trait evolution models. Syst. Biol 63:397–408.

Howard, C.C., R.A. Folk, J.M. Beaulieu, N. Cellinese. 2019. The monocotyledonous underground: global climatic and phylogenetic patterns of geophyte diversity. Am. J. Bot 106:850–863.

Ives, A.R., T. Garland. 2010. Phylogenetic logistic regression for binary dependent variables. Syst. Biol 59:9–26.

R Core Team. 2022 R: A language and environment for statistical computing. Vienna, Austria.

Zeng, Y., C. O'Malley, S. Singhal, F. Rahim, S. Park, X. Chen, R. Dudley. 2020. A tale of winglets: evolution of flight morphology in stick insects. Front. Ecol. Evol 8:121.

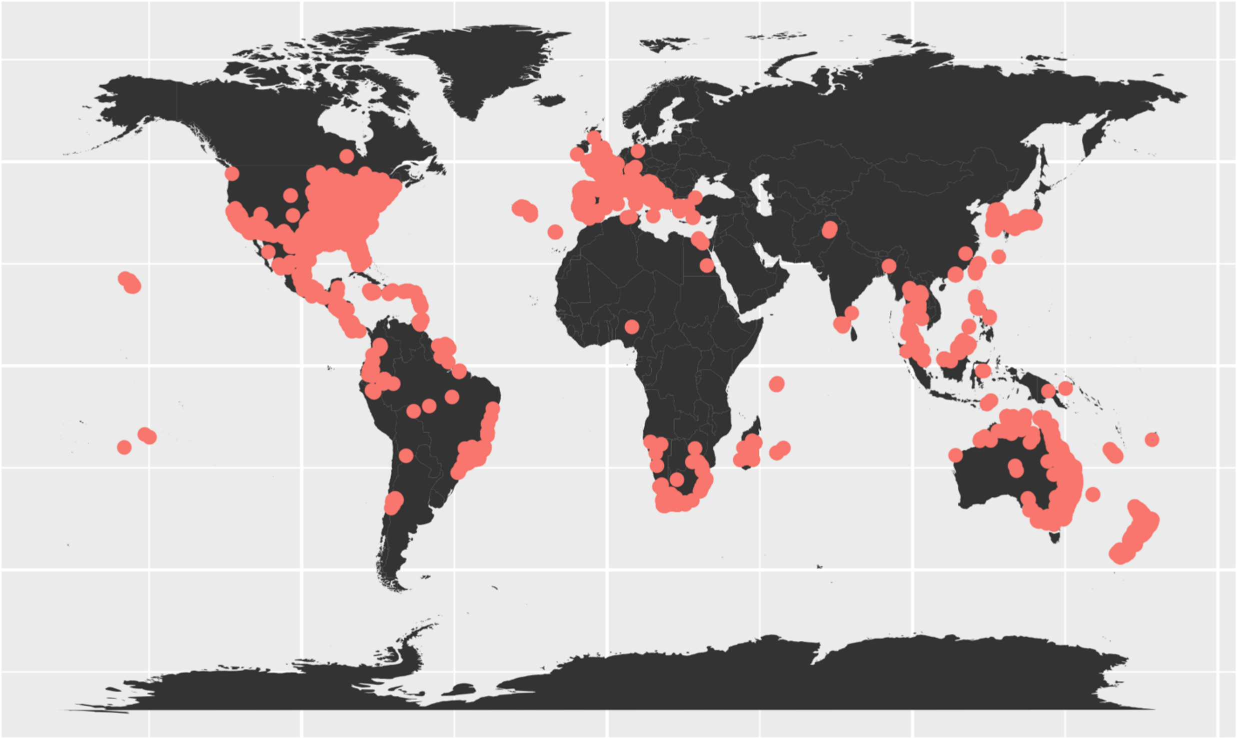

**Figure S1.** Map showing the geographic distribution of the individuals used in this study.

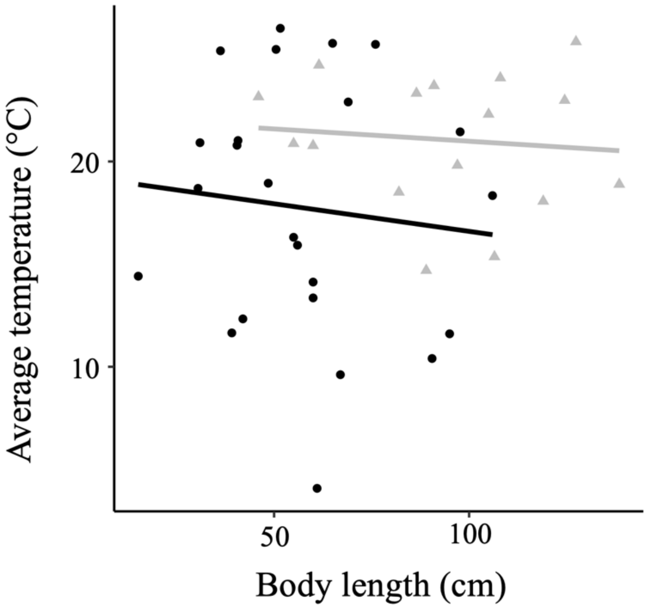


**Figure S2.** Flight capable species (gray triangles) are found in warmer environments compared to species that are flightless (black circles), even when taking differences in body size into consideration. This figure does not correct for phylogeny.

**Table S1.** The lack of an association between windspeed and flying ability is consistent, no matter the minimum number of occurrences that are required or the phylogeny that is used.

| Minimum number of occurrences | Number of species | Phylogeny | Estimated Coefficient | z | P |
|---|---|---|---|---|---|
| 20 | 39 | B1 | -0.515 | -1.380 | 0.168 |
| | | B2 | -0.545 | -1.479 | 0.139 |
| | | B3 | -0.543 | -1.477 | 0.140 |
| 10 | 59 | B1 | -0.318 | -1.124 | 0.261 |
| | | B2 | -0.327 | -1.148 | 0.251 |
| | | B3 | -0.309 | -1.100 | 0.271 |
| 3 | 107 | B1 | -0.017 | -0.093 | 0.927 |
| | | B2 | -0.035 | -0.189 | 0.850 |
| | | B3 | -0.011 | -0.063 | 0.950 |

**Table S2.** Even when taking elevation into consideration, temperature is positively associated with flying ability in phasmids.

| Variable | Estimated Coefficient | z | P |
|---|---|---|---|
| Elevation | 0.001 | 1.643 | 0.100 |
| Temperature | 0.202 | 3.379 | 0.001 |

**Table S3.** Even when taking elevation into consideration, windspeed is not associated with flying ability in phasmids.

| Variable | Estimated Coefficient | z | P |
|---|---|---|---|
| Elevation | -0.001 | -1.461 | 0.144 |
| Windspeed | -0.208 | -1.210 | 0.226 |
